# IgA Vasculitis Presenting as Pulmonary-Renal Syndrome

**DOI:** 10.7759/cureus.77111

**Published:** 2025-01-07

**Authors:** José Mário Bastos, Joana Medeiros, Catarina Oliveira Silva, Johanna Viana, Sofia Marques

**Affiliations:** 1 Nephrology, Unidade Local de Saúde de Braga, Braga, PRT

**Keywords:** alveolar hemorrhage, glomerulonephritis, iga vasculitis, plasmapheresis, pulmonary-renal syndrome, small-vessel vasculitis

## Abstract

Immunoglobulin A (IgA) vasculitis (IgAV), formerly known as Henoch-Schönlein purpura, is a small-vessel vasculitis characterized by the deposition of IgA-containing immune complexes in vessel walls. While predominantly affecting the pediatric population, adult-onset IgAV often presents with a more severe clinical course and a higher risk of renal complications. Pulmonary-renal syndrome, characterized by diffuse alveolar hemorrhage and glomerulonephritis, is an exceedingly rare manifestation of IgAV and poses substantial diagnostic and therapeutic challenges.

We report a case of a 33-year-old male presenting with hemoptysis, fatigue, and diffuse myalgias. The patient had no cutaneous manifestations, such as purpura. Laboratory workup revealed anemia, acute kidney injury with proteinuria, and microscopic hematuria, alongside negative serologies for antineutrophil cytoplasmic antibodies (ANCA), anti-glomerular basement membrane antibodies (anti-GBM), and other autoimmune markers. A chest computed tomography (CT) scan demonstrated bilateral ground-glass opacities, consistent with diffuse alveolar hemorrhage, which was later confirmed by bronchofibroscopy. Renal biopsy confirmed IgA vasculitis nephritis, with mesangial hypercellularity, segmental sclerosis, and dominant IgA deposition.

Pulmonary-renal syndrome secondary to IgAV was assumed, and the patient was treated with high-dose intravenous methylprednisolone, followed by oral prednisolone, alongside plasma exchange. This led to the resolution of hemoptysis and the recovery of renal function. Sustained remission was achieved with careful tapering of corticosteroids over the subsequent year.

This case underscores the importance of a high index of suspicion and a multidisciplinary approach to managing IgAV, particularly in atypical presentations involving pulmonary-renal syndrome. Early diagnosis and aggressive immunosuppressive therapy are essential to achieving favorable outcomes, even in severe cases.

## Introduction

Immunoglobulin A (IgA) vasculitis (IgAV), previously known as Henoch-Schönlein purpura, is a systemic vasculitis characterized by the deposition of immunoglobulin A (IgA)-containing immune complexes in the walls of small blood vessels. It predominantly affects the skin, gastrointestinal tract, joints, and kidneys, leading to a wide spectrum of clinical manifestations. Although 90% of cases occur in the pediatric population, IgAV can also present in adults, often with a more severe clinical course and a higher risk of renal involvement [1,2].

The pathogenesis of IgAV involves the formation of galactose-deficient IgA1 immune complexes, which trigger complement activation and vascular inflammation. Environmental factors, genetic predisposition, and abnormal immune responses are thought to contribute to the disease, although the exact mechanisms remain incompletely understood [3,4].

Renal involvement, referred to as IgAV nephritis, is one of the most significant complications of IgAV and a major determinant of long-term prognosis. It typically presents as hematuria, proteinuria, and, in severe cases, nephrotic syndrome or rapidly progressive glomerulonephritis. Histologically, it is indistinguishable from IgA nephropathy, with mesangial deposition of IgA-dominant immune complexes seen on kidney biopsy [4]. Renal manifestations can occur in up to 50% of adult patients and, compared to pediatric cases, carry a higher risk of progression to chronic kidney disease and end-stage renal disease (ESRD) [5].

In rare cases, IgAV can present with pulmonary-renal syndrome, a severe and potentially life-threatening condition characterized by diffuse alveolar hemorrhage and glomerulonephritis. Pulmonary hemorrhage is an uncommon manifestation of IgAV and poses substantial diagnostic and therapeutic challenges, particularly in patients without cutaneous purpura, a hallmark clinical feature of the disease [3].

## Case presentation

We describe the case of a 33-year-old male with no significant medical history or use of chronic medication who presented to the emergency department with hemoptysis. The patient also reported fatigue and diffuse myalgias, which lasted approximately two weeks. He denied any decrease in urinary output, macroscopic changes in urine, recent infections, or drug exposures.

On physical examination, the patient appeared pale but hydrated, conscious, and cooperative. Vital signs revealed tachycardia (heart rate of 112 bpm), hypertension (blood pressure of 155/98 mmHg), and an axillary temperature of 36.8°C. Peripheral oxygen saturation was 97% on room air, and lung auscultation revealed scattered bilateral crackles. Bilateral axillary lymphadenopathies with inflammatory characteristics (tender, soft, and mobile) were noted. No rash, purpura, or other cutaneous changes were observed. Cardiovascular and abdominal examinations were otherwise unremarkable.

**Figure 1:**
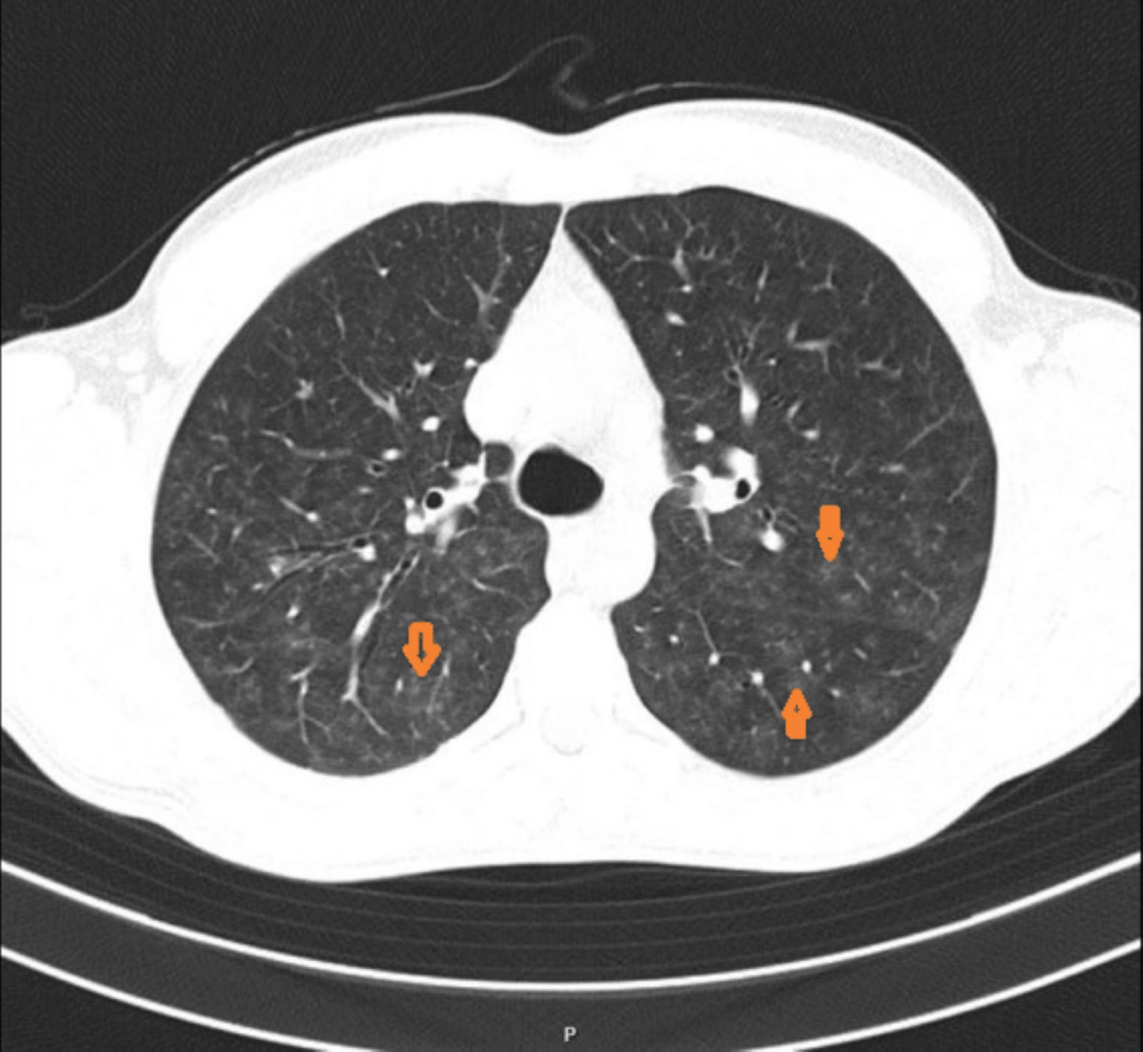
Chest computed tomography (CT) scan showing bilateral ground-glass opacities (orange arrows), consistent with alveolar hemorrhage.

Initial laboratory results showed anemia, with a hemoglobin level of 6.8 g/dL, and acute kidney injury (AKI) with a creatinine level of 4.25 mg/dL (baseline of 0.8 mg/dL two months prior). Urine sediment analysis revealed significant microscopic hematuria (>50 RBCs/high-power field), with a urinary protein-to-creatinine ratio (UPCR) of 2.8 g/g and a urinary albumin-to-creatinine ratio (UACR) of 1600 mg/g, which was subsequently confirmed by a 24-hour urine collection showing proteinuria of 3400 mg and albuminuria of 1900 mg. Serologic testing for antineutrophil cytoplasmic antibodies (ANCA), anti-globular basement membrane antibodies (anti-GBM), antinuclear antibodies (ANA), and anti-double-stranded DNA antibodies (anti-dsDNA) was negative, and complement levels were normal. Serum IgA levels were elevated (448 mg/dL for a normal range of 40-350 mg/dL). Protein immunoelectrophoresis showed no monoclonal peaks. Screening for HIV, hepatitis B, hepatitis C, and syphilis was also negative. A chest computed tomography (CT) scan demonstrated bilateral, scattered ground-glass opacities consistent with alveolar hemorrhage (Figure 1).

The patient was admitted to the nephrology department for further evaluation and management. Daily plasma exchange therapy was initiated, with a total replacement volume of 4.5 L of fresh frozen plasma, calculated based on the patient's weight (84 kg) and hematocrit (23.7%). High-dose intravenous methylprednisolone (1000 mg daily for three days) was administered. Hemoptysis had ceased by the third day of hospitalization, and plasma exchange was discontinued. The patient was transitioned to oral prednisolone at a dose of approximately 1 mg/kg/day (80 mg/day).

**Figure 2:**
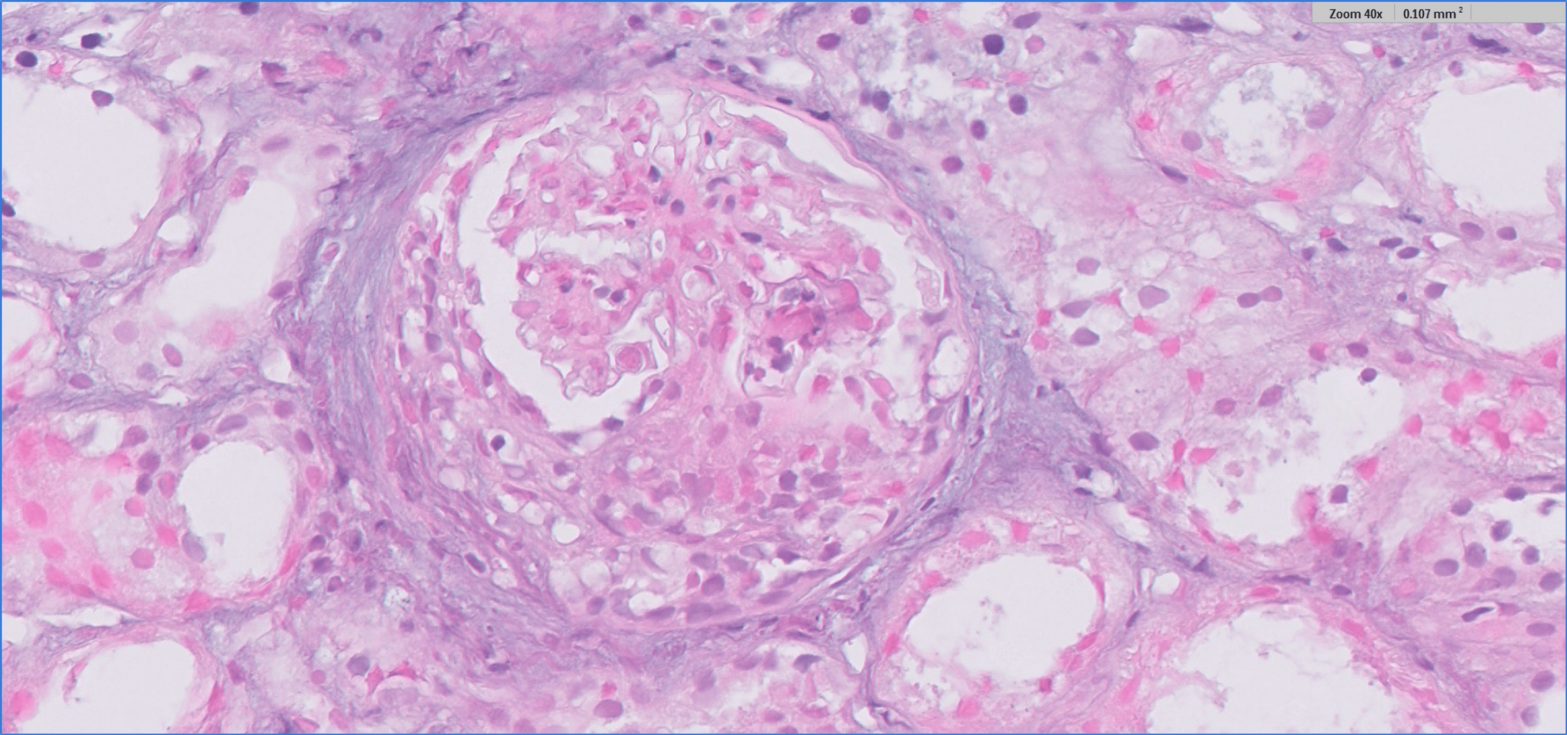
Renal biopsy showing a glomerulus with a fibrocellular crescent. Periodic acid-Schiff (PAS) stain, magnification: ×40.

**Figure 3:**
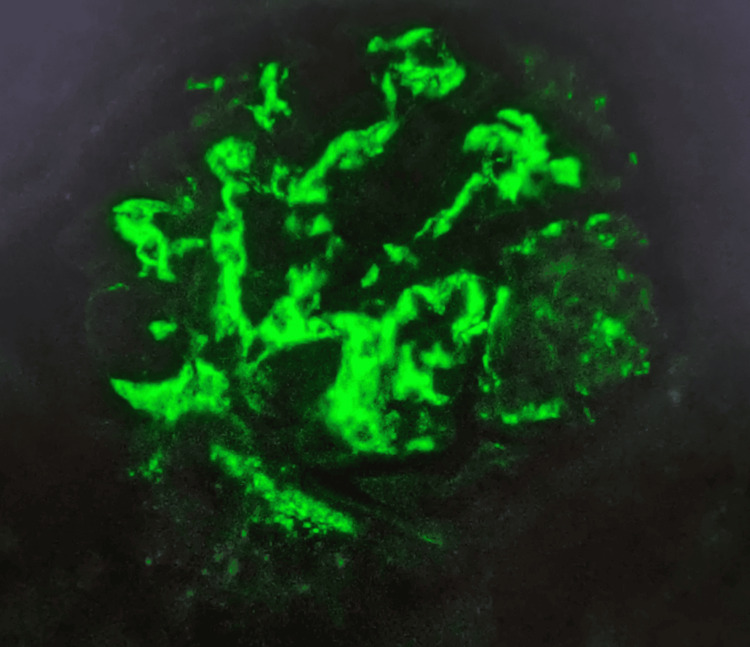
Immunofluorescence showing dominant mesangial deposition of IgA. IgA-specific fluorescein isothiocyanate staining, magnification: ×40.

A renal biopsy was performed, revealing 24 glomeruli, of which three showed segmental sclerosis and three exhibited fibrocellular crescents (Figure 2). Mild mesangial hypercellularity (Oxford M1) and mild interstitial nephritis were noted, with no significant tubular atrophy or interstitial fibrosis (T0). Immunofluorescence (Figure 3) demonstrated dominant mesangial deposition of IgA (+++), with co-deposition of C3 (++). Trace amounts of IgM and IgG (+) were also observed. These findings were consistent with IgAV nephritis (Oxford classification: M1 E0 S1 T0 C1).

A bronchofibroscopy, conducted in collaboration with the pulmonology team, confirmed alveolar hemorrhage with bronchoalveolar lavage, revealing rosy fluid containing hemosiderin-laden macrophages.

During hospitalization, under corticosteroid therapy, close monitoring showed resolution of hematuria, gradual improvement in proteinuria, and recovery of kidney function. Two months post-presentation, the patient was asymptomatic, with proteinuria reduced to 1200 mg/24 hours, albuminuria to 700 mg/24 hours, a urinary sediment without hematuria (<5 RBCs/high-power field), and a creatinine level of 1.0 mg/dL.

The patient continued a tapering course of prednisolone, which was gradually reduced and definitively discontinued 10 months after initiation. At the outpatient follow-up one year after presentation, the patient remained asymptomatic, with stable renal function (serum creatinine 0.9 mg/dL), no hematuria, a UPCR of 0.1 mg/mg, a UACR of 0.07 mg/mg, and no recurrence of alveolar hemorrhage.

## Discussion

This case emphasizes the complexity of diagnosing and managing IgAV when it presents with atypical features. The absence of cutaneous purpura, a hallmark of the disease, posed significant diagnostic challenges. Instead, the patient's presentation with hemoptysis, anemia, and AKI was indicative of pulmonary-renal syndrome, a rare but life-threatening manifestation of IgAV. The chest CT scan showed bilateral ground-glass opacities, consistent with diffuse alveolar hemorrhage, which was later confirmed by bronchofibroscopy, demonstrating hemosiderin-laden macrophages. The diagnosis of IgAV-related alveolar hemorrhage was further supported by negative serologic markers for ANCA, anti-GBM, and other autoimmune antibodies [6,7].

Although alveolar hemorrhage is more commonly linked to anti-GBM disease and ANCA-associated vasculitis, it can occur in IgAV [8,9]. A retrospective review conducted at the Mayo Clinic over six years identified pulmonary involvement in 2.4% of 124 adult patients with IgAV [10]. In a systematic review of 36 cases of diffuse alveolar hemorrhage in IgAV, common features included hemoglobin drop (74%), hemoptysis (75%), and chest infiltrates (94%), with approximately half of the patients requiring mechanical ventilation due to delayed recognition [11].

The pathogenesis of pulmonary-renal syndrome in IgAV remains unclear but is hypothesized to involve IgA-mediated immune injury to pulmonary capillaries, mirroring the mechanism observed in renal involvement. It demands a high index of suspicion, especially when classical features like purpura are absent. Timely recognition and diagnosis are crucial to prevent complications and initiate appropriate treatment [6,7].

The renal presentation in this case was consistent with nephritic syndrome, characterized by hypertension, hematuria, proteinuria, and AKI. Renal biopsy findings confirmed IgAV nephritis, revealing mesangial hypercellularity with dominant IgA deposition, segmental sclerosis, and fibrocellular crescents. These findings, classified as M1 E0 S1 T0 C1 under the Oxford classification, suggest an intermediate risk of renal progression [12,13]. Adults with IgAV nephritis, particularly those with crescents or significant proteinuria, face an increased risk of developing ESRD. Proteinuria exceeding 1 g/day, as seen in this case, is a key marker of disease severity and a critical target for therapeutic intervention [8,14].

The therapeutic approach for this patient complied with established guidelines for severe IgAV nephritis and pulmonary hemorrhage. Aggressive immunosuppression with high-dose intravenous methylprednisolone followed by oral prednisolone effectively controlled systemic inflammation and prevented further organ damage [12,13]. The early use of plasma exchange was instrumental in removing circulating immune complexes and stabilizing acute lung and kidney injuries [13].

The patient's response to treatment was favorable, with resolution of hemoptysis, improvement of anemia, and recovery of kidney function during hospitalization. The subsequent reduction in proteinuria and normalization of urinary sediment pointed out the efficacy of corticosteroids in managing IgAV nephritis. Sustained remission during long-term follow-up highlights the importance of careful corticosteroid tapering to minimize relapse risk while avoiding adverse effects [14].

## Conclusions

This case stands out for its atypical presentation of IgAV in an adult patient, manifesting as pulmonary-renal syndrome with diffuse alveolar hemorrhage and nephritic syndrome in the absence of classical cutaneous features, such as purpura. It also highlights the broad spectrum of differential diagnoses a nephrologist must consider when evaluating patients with AKI of glomerular origin.

Ultimately, this case demonstrates that a high index of suspicion, timely diagnosis, aggressive immunosuppressive therapy, and a multidisciplinary approach are essential to achieving favorable outcomes, even in severe and atypical presentations of IgAV.
